# Spread of GES-5 carbapenemase-producing *Pseudomonas aeruginosa* clinical isolates in Japan due to clonal expansion of ST235

**DOI:** 10.1371/journal.pone.0207134

**Published:** 2018-11-19

**Authors:** Tomomi Hishinuma, Tatsuya Tada, Kyoko Kuwahara-Arai, Norio Yamamoto, Masahiro Shimojima, Teruo Kirikae

**Affiliations:** 1 Department of Microbiology, Juntendo University School of Medicine, Tokyo, Japan; 2 BML Inc., Kawagoe, Saitama, Japan; Zhejiang University, CHINA

## Abstract

The first outbreak in Japan of GES-5 carbapenemase-producing *Pseudomonas aeruginosa* occurred in a long-term care facility in 2014. To assess the spread of GES-5 producing *P*. *aeruginosa* clinical isolates in medical settings in Japan, 1,476 carbapenem-resistant *P*. *aeruginosa* isolates obtained from 2012 to 2016 were characterized. Of these 1,476 isolates, 104 (7.0%) harbored *bla*_GES-5_. Southern blotting revealed that the *bla*_GES-5_ was located on the chromosome. The isolation rates of these GES-5 producers increased significantly every year, from 2.0% (6 of 295) in 2012 to 2.8% (8 of 283) in 2013 to 5.3% (16 of 303) in 2014 to 9.7% (29 of 300) in 2015 to 15.3% (45 of 295) in 2016. Of the 104 GES-5 producers, 102 belonged to clonal complex (CC) 235, including 99 belonging to ST235 and three belonging to ST2233). Whole genome sequence analysis revealed that CC235 *P*. *aeruginosa* harboring *bla*_GES-5_ spread in a clonal manner. These results indicate that these GES-5 producing CC235 *P*. *aeruginosa* clinical isolates have spread in medical settings throughout Japan.

## Introduction

The dissemination of carbapenem-resistant *Pseudomonas aeruginosa*, known as *P*. *aeruginosa* high-risk clones, has become a serious problem in medical settings worldwide [1,2]. GES-type β-lactamases, belonging to class A extended-spectrum β-lactamases (ESBLs) [3], have been increasingly reported among Gram-negative pathogens, including *P*. *aeruginosa*, *Enterobacter cloacae*, *Klebsiella pneumoniae*, and *Acinetobacter baumannii* [4]. GES-1 was initially detected in a *K*. *pneumoniae* isolate in 1998 in France [5]. To date, 37 GES variants have been described (ftp://ftp.ncbi.nlm.nih.gov/pathogen/betalactamases/Allele.tab).

Some GES-type enzyme variants, including GESs-2, -4, -5, -6 and -14, have shown carbapenem-hydrolyzing activities [6–10]. GES-2 was the first GES-type enzyme with carbapenem-hydrolyzing activities to be identified, although its activities differ from GES-1 by a substitution of Gly170Asn, located inside the Ω-loop of the catalytic site [11]. GESs-4, -5, -6, and -14, with a substitution of Gly170Ser, exhibit higher carbapenem-hydrolyzing activities than GES-1, and GES-5 exhibit higher carbapenem-hydrolyzing activities than GES-5 [6–10].

ST235 multidrug-resistant *P*. *aeruginosa* co-producing IMP-type metallo-β-lactamases and AAC(6’)s was originally isolated in 2003 in Japan [12], and it was a cause of outbreaks in community hospitals throughout Japan [13]. The first outbreak of GES-5 producing *P*. *aeruginosa* in Japan occurred in 2014 [14]. The objective of this study was to characterize the spread of carbapenem-resistant GES-5 producing *P*. *aeruginosa* in Japan by analyzing 1476 clinical isolates obtained from 2012 to 2016.

## Materials and methods

### Bacterial strains

*P*. *aeruginosa* clinical isolates identified as carbapenem-resistant were isolated from 1,476 individual patients at hospitals located in all 47 prefectures throughout Japan between 2012 and 2016 (295 in 2012, 283 in 2013, 303 in 2014 300 in 2015 and 295 in 2016) in hospitals by BML Biomedical Laboratories R&D Center (Kawagoe, Saitama, Japan). Drug-susceptibility was tested using the microdilution method according to the criteria of the Clinical Laboratory Standards Institute (CLSI) criteria, with carbapenem-resistant *P*. *aeruginosa* defined as isolates resistant to imipenem or meropenem (MIC ≥ 8 μg/mL) [15].

### Detection and sequencing of GES-type ESBLs

Carbapenem-resistant *P*. *aeruginosa* isolates were screened for *bla*_GESs_-type genes by PCR using the primers GES-F (5’-ATGCGCTTCATTCACGCAC-3’) and GES-R (5’-CTATTTGTCCGTGCTCAGG-3’), as described [16]. All PCR products were sequenced using a DNA sequencer (ABI PRISM 3130; Applied Biosystems, Foster City, CA).

### Whole genome sequencing

Genomic DNAs of *bla*_GES-5_-positive isolates were extracted using DNeasy Blood and Tissue kits (Qiagen, Tokyo, Japan) and sequenced by a next generation sequencer (MiSeq; Illumina, San Diego, CA).

### Phylogenetic analysis

To identify single nucleotide polymorphisms (SNPs) throughout the entire genomes of all 104 *bla*_GES-5_-positive isolates, all reads of each isolate were aligned against *P*. *aeruginosa* NCGM2.S1 (Gen Bank accession no. AP 012280) using CLC genomics workbench version 8.0.2 (CLC bio, Tokyo, Japan) [17]. SNP concatenated sequences were aligned using MAFFT (http://mafft.cbrc.jp/alignment/server/). Models and parameters used for the phylogenetic analyses were computed using j-Model Test-2.1.4. A maximum-likelihood phylogenetic tree was constructed from SNP alignment with PhyML 3.0 [18].

### Drug resistance genes and MLST

Genes associated with resistance to β-lactams, aminoglycosides and quinolones were detected using ResFinder 3.0 (https://cge.cbs.dtu.dk/services/ResFinder/). Fluoroquinolone resistance has been associated with mutations in the quinolone resistance determining region, which includes the *gyrA* and *parC* genes that encode DNA gyrase and topoisomerase IV, respectively [19]. Multilocus sequence types (MLSTs) were deduced as described (http://pubmlst.org/paeruginosa/). Clonal complexes (CC) were determined by eBURST version 3 (http://eburst.mlst.net).

### Pulsed-field gel electrophoresis and Southern blotting

DNA plugs of two *bla*_GES-5_ positive CC235 isolates, NCGM2108 (ST235) and NCGM2900 (ST2233), were digested with S1 nuclease, separated by pulsed-field gel electrophoresis (PFGE), and subjected to Southern blotting and hybridization using *bla*_GES-5_ probes.^13^

### Statistical analysis

The yearly proportions of multidrug-resistant *P*. *aeruginosa* isolates positive for *bla*_GES-5_ were analyzed by the chi-square test.

### Nucleotide sequence accession numbers

The whole genome sequences of all 104 *bla*_GES-5_-positive isolates have been deposited in GenBank as Accession Number DRA006450 (https://www.ncbi.nlm.nih.gov/sra/?term=DRA006450) (NCGM2012-2015) and DRA007009 (NCGM2016). The sequences of the genomic environment surrounding *bla*_GES-5_ in multidrug-resistant *P*. *aeruginosa* strains NCGM2100 (ST274), NCGM2900 (ST235) and NCGM3294 (ST1342) have been deposited in GenBank as Accession Numbers LC157846, LC155936 and LC360798, respectively.

## Results

### Identification and drug susceptibilities of *bla*_GES-5_-positive *P*. *aeruginosa* isolates

**Fig 1:**
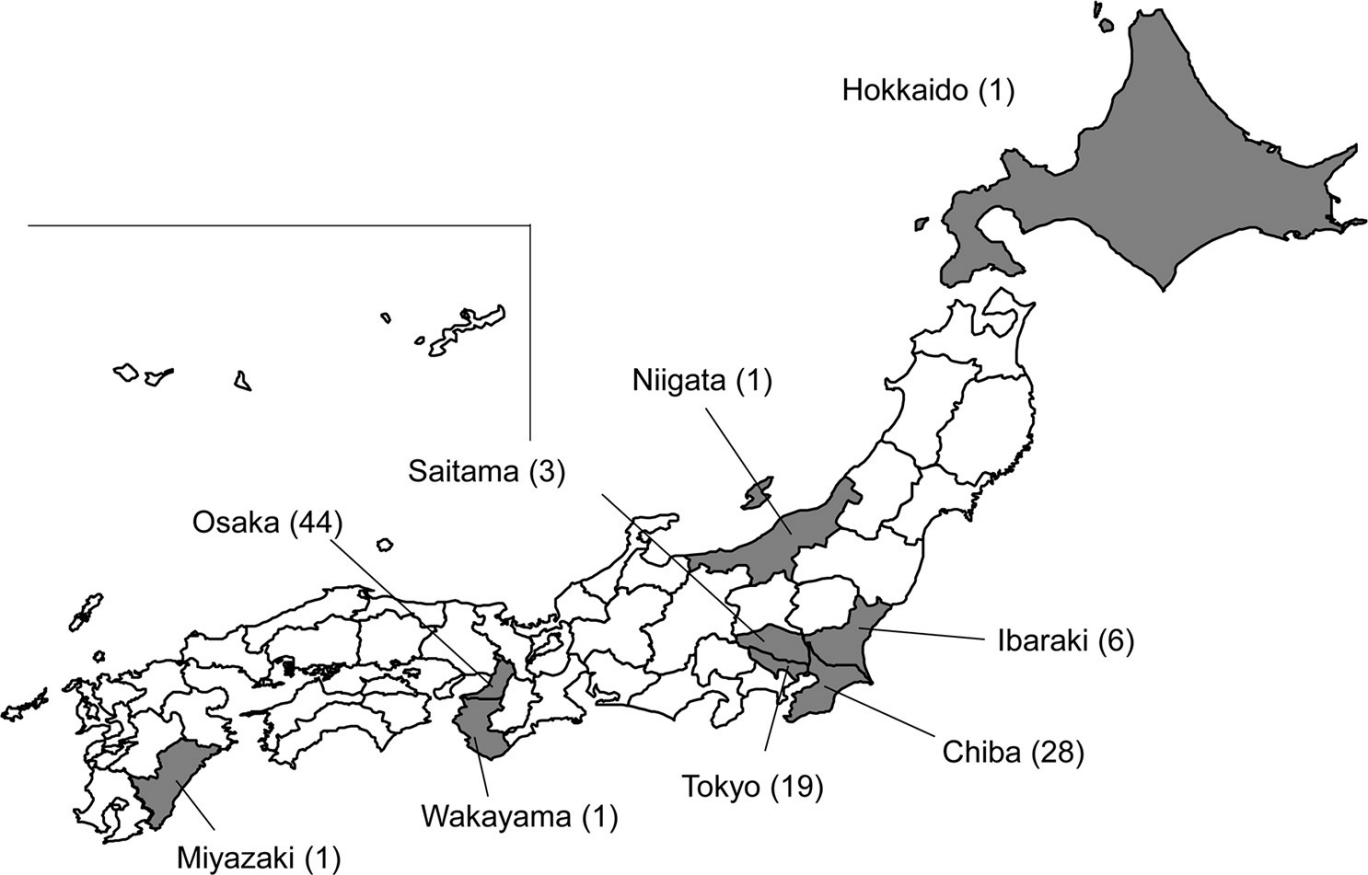
Geographical distribution of the nine prefectures from which the *bla*_GES-5_-containing *P*. *aeruginosa* strains were isolated. The numbers of the isolates in each prefecture were indicated in parentheses.

Of 1,476 carbapenem-resistant isolates positive *P*. *aeruginosa* isolates, 137 (9.3%) were positive for *bla*_GESs_. Sequence analysis revealed that, of these isolates, four (2.9%) were positive for *bla*_GES-1_, 104 (75.9%) for *bla*_GES-5_, two (1.5%) for *bla*_GES-6_, five (3.6%) for *bla*_GES-15_, one (0.7%) for *bla*_GES-24_, 15 (10.9%) for *bla*_GES-26_, one (0.7%) for *bla*_GES-29_, three (2.2%) for *bla*_GES-30_, and two (1.5%) for a novel variant of *bla*_GES_ (GenBank accession no. LC385763). The proportion of *bla*_GES-5_-positive isolates increased significantly by year, being 2.0% (6/295) in 2012, 2.8% (8/283) in 2013, 5.3% (16/303) in 2014, 9.7% (29/300) in 2015 and 15.3% (45/295) in 2016 (*p* ≤0.01 for 2016 vs 2012). These 104 *bla*GES-5-positive isolates were obtained in nine regions of Japan, including 44 (42%) were in Osaka, 28 (27%) in Chiba, 19 (18%) in Tokyo, six (6%) in Ibaraki, three (3%) in Saitama, and one each (1.0%) in Hokkaido, Niigata, Wakayama, and Miyazaki (Fig 1). Of these isolates, 71 (68.3%) were from respiratory tracts, 22 (21.2%) from urinary tracts, seven (6.7%) from decubitus and four (3.8%) from pus.

All 104 of these *bla*_GES-5_-positive isolates were resistant to imipenem and meropenem but sensitive to colistin (Table 1). Both MIC_50_ and MIC_90_ values were higher for meropenem than for imipenem. In addition, 50 isolates were resistant to amikacin, 28 to aztreonam, 19 to cefepime, 34 to ceftazidime and 103 to ciprofloxacin.

**Table 1:** Characterization of GES-5 producing *P*. *aeruginosa* isolates, including MIC ranges, MLST, drug-resistant factors and virulent factors.

MLST	No. of isolates^a^	MIC Range against antibiotics^b^ (mg/L) (% resistance)^c^								Carbapenemase- and ESBL- encoding gene(s)	Aminoglycoside resistance gene(s)	Mutation in DNA gyrase		Exotoxin gene(s)
		AMK	AZT	FEP	CAZ	CIP	CST	IMP	MEM			GyrA	ParC	
ST235 and ST2233 (CC235)	102	4–64 (47%)	2–64 (25%)	4–128 (17%)	4–512 (31%)	2–64 (99%)	0.063–0.5 (0%)	16–64 (100%)	32–512 (100%)	*bla*_GES-5_ *bla*_PAO_ *bla*_OXA-488_	*aac(6')-Ib*	T83I	S87L	*exoT* *exoU* e*xoY*
ST274	1	128	128	64	128	32	0.5	64	64	*bla*_GES-5_ *bla*_PAO_ *bla*_OXA-486_	*aac(6')-Ia*	T83I	S87L	*exoS* *exoT* e*xoY*
ST1342	1	64	64	32	64	32	0.5	64	128	*bla*_GES-5_ *bla*_PAO_ *bla*_OXA-396_ *bla*_CMY-8_	*aac(6')-Ia*	T83I	S87L	*exoS* *exoT* e*xoY*

^a^Of 102 isolates belonging to CC235, 99 isolates belonged to ST235 and the remaining 3 did to ST2233.

^b^AMK: Amikacin, AZT: Aztreonam, FEP: Cefepime, CAZ: Ceftazidime, CIP: Ciprofloxacin, CST: Colistin, IMP: Imipenem, MEM: Meropenem.

^c^Breakpoints for resistance (mg/L): AMK; ≥32, AZT; ≥32, FEP; ≥32, CAZ; ≥32, CIP; ≥4, CST; ≥8, IMP; ≥8, and MEM; ≥8.

All 104 isolates harbored *bla*_PAO._ Of them, 102 (98.0%) harbored *bla*_OXA-488_, 1 had *bla*_OXA-396_ and 1 had *bla*_OXA-486_ (Table 1). Of the 104 *bla*_GES-5_-positive isolates, 102 (98.0%) harbored *aac(6’)-Ib* and the other two harbored *aac(6’)-Ia* (Table 1). The all 104 isolates were found to have point mutations in the quinolone-resistance-determining regions of *gyrA* and *parC*, consisting of the amino acid substitutions S83I in GyrA and S87L in ParC (Table 1). Of them, a quinolone-sensitive strain had the same mutations as the remaining quinolone-resistant strains, but it had amino acid substitutions in the efflux systems, including P363L in OprJ, R112Q in OprN, in addition to an OprD in-frame deletion. In-frame deletions of OprD were found in 54 of 104 isolates tested. The 99 isolates belonging to CC235 (ST235 and ST2233) harbored virulence genes, *exoT*, *exoU* and *exoY*, although the remaining two belonging ST274 and ST1342 did *exoS*, *exoT* and *exoY* (Table 1).

### MLST and phylogenetic analysis

**Fig 2:**
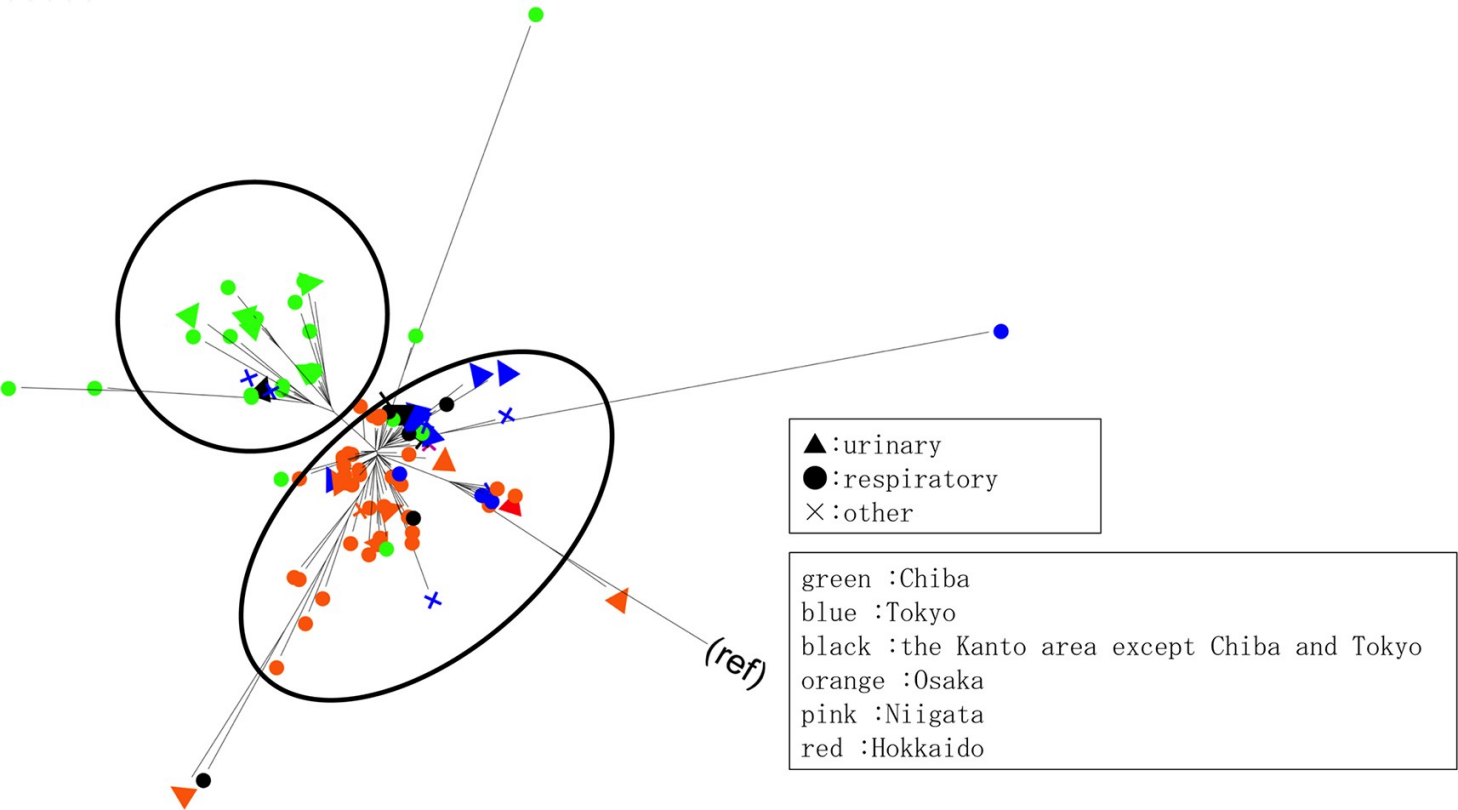
Molecular phylogeny of the 102 *P*. *aeruginosa* strains belonging to CC235. A maximum-likelihood phylogenetic tree was constructed from these isolates. The GTR+I+G model was chosen as a nucleotide substitution model.

Of the 104 *bla*_GES-5_-positive isolates, 102 belonged to Clonal Complex (CC) 235, including 99 and 3 belonging to ST235 and ST2233, respectively. The individual isolates from Miyazaki and Wakayama prefectures belonged to ST274 (allelic profile: 23, 5, 11, 7, 1, 12, 7) and ST1342 (allelic profile: 1, 5, 26, 3, 1, 10, 3), respectively, and were unrelated to CC235. A maximum-likelihood phylogenetic tree constructed from the 102 CC235 isolates showed two major clades (Fig 2). Clade A mainly consisted of isolates from Hokkaido, Ibaraki, Niigata, Osaka, Saitama and Tokyo, whereas clade B mainly consisted of isolates from Chiba (Figs 1 and 2).

### Genomic environments surrounding *bla*_GES-5_

**Fig 3:**
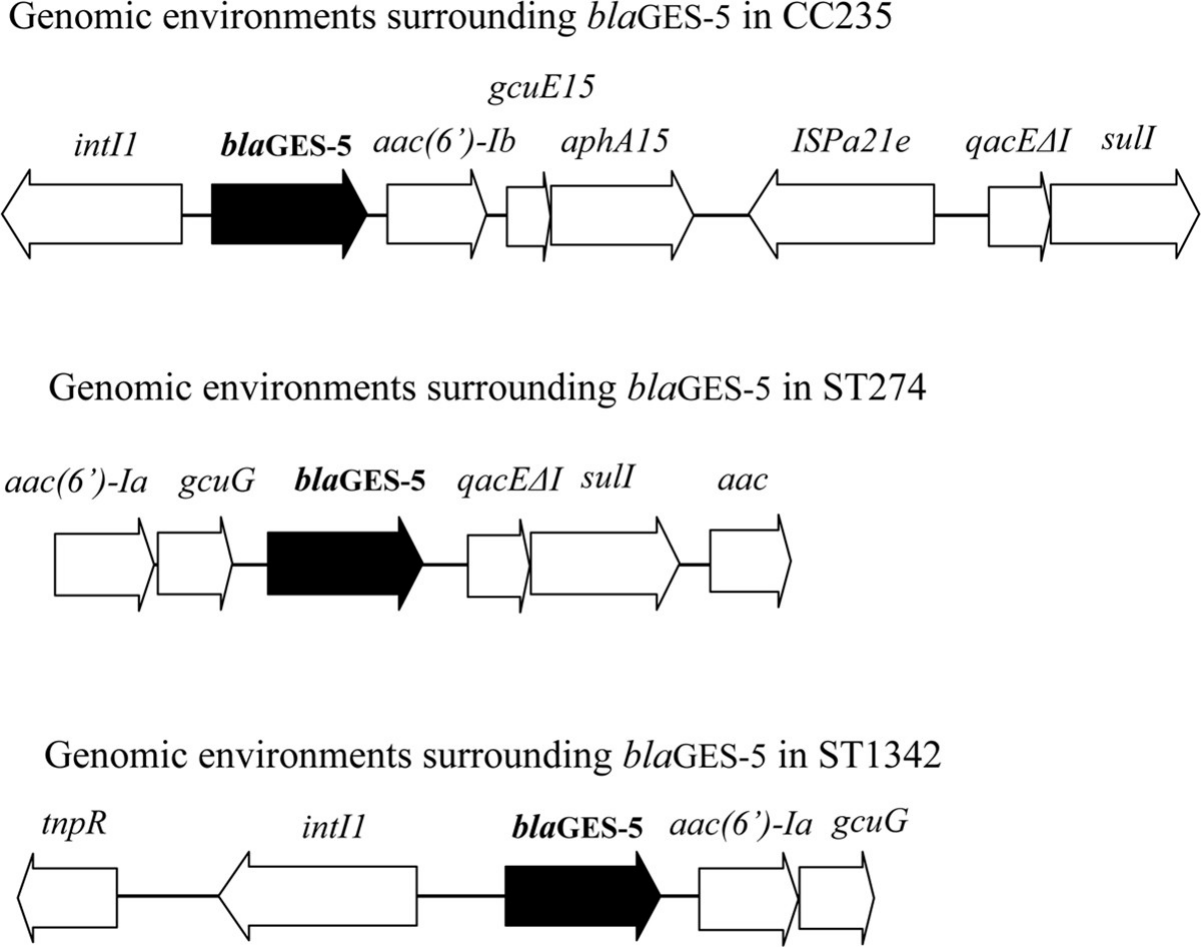
Structure of the genomic environments surrounding *bla*_GES-5_.

In the 102 isolates belonging to CC235, *bla*_GES-5_ was present in a class 1 integron (accession no. LC155936) (Fig 3). The sequence of this integron from nt 1 to nt 6,647 was identical to the sequence from nt 10,786 to nt 17,432 of *P*. *aeruginosa* strain CH79 (C79) fosmid2CA-integron isolated in 2010 in Sydney, Australia (accession no. JF826499) [20]. The genomic environment surrounding *bla*_GES-5_ in the ST274 isolate from Miyazaki was *aac(6’)-Ia*-*gcuG-bla*_GES-5_*- qacEΔ1-sulI-aac* (accession no. LC157846) (Fig 3). The sequence of this genomic environment from nt 140 to nt 1,127, which contained *aac(6’)-Ia*-*gcuG*, was identical to the sequence from nt 980 to nt 1,967 of integron In831 in *P*. *aeruginosa* TUM4030 isolated in 2004 in Hokkaido, Japan (accession no. AB901039) [21], and the sequence from nt 1,297 to nt 4,135, containing the structure *bla*_GES-5_*-qacEΔ1-sulI-aac*, was more than 99% identical to the sequence from nt 18,969 to nt 21,807 of a plasmid pGES5 in *Aeromonas hydrophilia* WCHAH01 isolated in China (accession no. KR014105). The genomic environment surrounding *bla*_GES-5_ in the ST1342 isolate from Wakayama was *tnpR*-*intI1-bla*_GES-5_*- aac(6’)-Ia*-*gcuG* (accession no. LC360798) (Fig 3). The sequence of the genomic environment from nt 100 to nt 9,341, which contained *tnpA-tnpR-intI1- bla*_GES-5_, was identical to the sequence from nt 2,314 to nt 12,842 of *P*. *aeruginosa* CH79 fosmid2CA-integron (accession no. JF826499) [20], and the sequence from nt 9,499 to nt 10,486, which contained *aac(6’)-Ia*-*gcuG*, was identical to the sequence from nt 980 to nt 1,967 of integron In831 in *P*. *aeruginosa* TUM4030 (accession no. AB901039) [21].

The 89,870 bp integrative conjugative element (ICE), including a class 1 integron with *bla*_GES-5_, was detected in CC235 GES-5 producing isolates by comparison with the sequence of PAO1 strain. The ICE was flanked by PA0747 (upstream region of the ICE) and PA2737 (downstream region of the ICE).

PFGE and Southern blotting showed that two *bla*_GES-5_-positive isolates belonging to CC235 (ST235 and ST2233) had no plasmid harboring *bla*_GES-5_ (data not shown).

## Discussion

This study showed that carbapenem-resistant *bla*_GES-5_-positive *P*. *aeruginosa* isolates, first isolated in Japan in 2012, rapidly spread throughout Japan over the next several years. Outbreaks of *bla*_GES-5_-positive isolates occurred among hospitals in a region (Chiba prefecture), indicating that *bla*_GES-5_-positive isolates started to expand in a clonal manner within a specific region in Japan. Our previous study on whole genome analysis of 136 clinical isolates of ST235 multidrug-resistant *P*. *aeruginosa* producing IMP-type MBLs showed that these isolates fell within seven subclades, with each subclade having a characteristic genetic background confined to a geographic location. The previous study indicated that *P*. *aeruginosa* ST235 has become prevalent worldwide due to the antibiotics-selective pressures through mutations and acquisition of resistant-elements among local populations [2]. Our results suggest that clonal expansion is the driving force in generating the population structure of ST235 *P*. *aeruginosa* [22].

The primary reason for the emergence and spread of carbapenem-resistant *bla*_GES-5_-positive *P*. *aeruginosa* isolates in medical settings in Japan is the inability of microbiological laboratories in Japan, even those in tertiary hospitals, to detect *bla*_GES-5_-positive isolates. *P*. *aeruginosa* strains that produce Class B metallo-β-lactamases can be routinely detected in microbiological laboratories using several methods [23–25]. These methods, however, are unable to detect bacterial strains producing class A carbapenemases. The cross-transmission of *bla*_GES-5_ will be caused by the clonal nature of the outbreaks in medical settings in Japan. Microbiological laboratories therefore require methods to easily detect GES-5 producers, such as the Blue Carba test [26,27], the CIMTris [28] and a systematic *bla*_GES_ PCR method.

Virulent factor ExoU will be associated with the antibiotic resistance and spreading of CC235 *P*. *aeruginosa*. Previous study reported that ExoU-positive *P*. *aeruginosa* strains had higher resistance to β-lactams and quinolones than ExoS-positive strains, because the ExoU-positive *P*. *aeruginosa* had more mutations in genes that were associated with β-lactams resistance and quinolone resistance [29]. The height of the mutation rate caused by ExoU may help to enhance the adaptability of CC235 *P*. *aeruginosa* to the environment.

GES-5 producing *P*. *aeruginosa* may spread in medical settings worldwide. A *P*. *aeruginosa* isolate producing GES-5 was first obtained in 2004 from the blood of a burn patient in China [16]. GES-5 producing *P*. *aeruginosa* strains have been isolated from patients in Brazil [30], Japan [14], Lithuania [31], South Africa [32], Spain [33] and Turkey [34]. Thus, it is important to monitor for GES-5 producing *P*. *aeruginosa* in medical settings worldwide.
